# Incorporating Pharmacometrics into Pharmacoeconomic Models: Applications from Drug Development

**DOI:** 10.1007/s40273-020-00944-0

**Published:** 2020-07-31

**Authors:** Meenakshi Srinivasan, Annesha White, Ayyappa Chaturvedula, Valvanera Vozmediano, Stephan Schmidt, Leo Plouffe, La’Marcus T. Wingate

**Affiliations:** 1 University of North Texas System College of Pharmacy, 3500 Camp Bowie Blvd, Fort Worth, TX 76107 USA; 2 Center for Pharmacometrics and Systems Pharmacology, College of Pharmacy, University of Florida, Orlando, FL USA; 3 Bayer HealthCare, Whippany, NJ USA; 4 College of Pharmacy, Howard University, Washington, DC USA

## Abstract

Pharmacometrics is the science of quantifying the relationship between the pharmacokinetics and pharmacodynamics of drugs in combination with disease models and trial information to aid in drug development and dosing optimization for clinical practice. Considering the variability in the dose–concentration–effect relationship of drugs, an opportunity exists in linking pharmacokinetic and pharmacodynamic model-based estimates with pharmacoeconomic models. This link may provide early estimates of the cost effectiveness of drug therapies, thus informing late-stage drug development, pricing, and reimbursement decisions. Published case studies have demonstrated how integrated pharmacokinetic–pharmacodynamic–pharmacoeconomic models can complement traditional pharmacoeconomic analyses by identifying the impact of specific patient sub-groups, dose, dosing schedules, and adherence on the cost effectiveness of drugs, thus providing a mechanistic basis to predict the economic value of new drugs. Greater collaboration between the pharmacoeconomics and pharmacometrics community can enable methodological improvements in pharmacokinetic–pharmacodynamic–pharmacoeconomic models to support drug development.

## Key Points for decision makers

**Table:** 

In addition to the current approach of generating and synthesizing evidence for estimating cost effectiveness, expanding the scope of pharmacoeconomic models by incorporating pharmacometric modeling and simulation-derived estimates for safety, efficacy, and effectiveness can provide value in drug development and clinical practice as shown in the published literature.
An opportunity exists for greater collaboration between clinical pharmacologists, pharmacoeconomists, and health outcomes researchers by leveraging the close alignment in their respective modeling methodologies to answer questions regarding cost-effectiveness and reimbursement decisions.

## Introduction

### Modeling and Simulation Approaches to Improve Drug Development Efficiency

The median costs incurred for bringing a new drug into the market is estimated to be US$985 million with return on investment for research and development expenditure at an all-time low for the pharmaceutical industry [1, 2]. Late-phase drug development accounts for the major portion of the costs with roughly half of investigational drugs reaching this phase failing because of safety and efficacy concerns or poor economic viability [3, 4]. Recognizing this, the US Food and Drug Administration (FDA) in its “critical path” initiative, acknowledged the opportunity of using modeling and simulation approaches to optimize and advance the drug development process and reduce costs [5]. Pharmacometrics is the science of quantifying the relationship between pharmacokinetics and pharmacodynamics of drugs in combination with disease models and trial information (specific target population, medication adherence) to aid in drug development and dosing optimization for clinical practice [6, 7]. Model-based drug development (MBDD), a framework built on Sheiner’s “learn and confirm” paradigm, implements these approaches across the drug development continuum using pharmaco-statistical models from preclinical and clinical data to evaluate the safety and efficacy of new drugs [8, 9]. Model-based drug development has been found to address root causes of drug failure; therefore, improving late-stage clinical development productivity, efficiency, and success [10]. Interested readers can refer to Table 1 for definitions of common pharmacometrics terminology used in this paper and find references to key tutorials. More recently, under the regulatory umbrella of the Prescription Drug User Fee Act VI, the US FDA has expanded MBDD to the model-informed drug development paradigm. Model-informed drug development has a range of applications including dose optimization, providing supportive evidence of efficacy using exposure–response analyses obtained through in silico approaches, and enabling policy development. Additionally, the Act provides a mechanism to facilitate discussion between drug developers and regulatory scientists to inform model-informed drug development in specific development programs [11].

**Table 1:** Definitions and tutorials papers of key terminology

Term	Brief definition	Key tutorial papers
Pharmacometrics	The analytical science using mathematical models to quantify the relationship between drug exposure and response for safety and efficacy, patient characteristics, disease progression, and clinical outcomes to make inferences for optimal drug dosing during drug development and clinical practice	[6, 17, 52]
Pharmacokinetic–pharmacodynamic modeling	Pharmacokinetic/pharmacodynamic modeling links the time course of drug absorption, distribution, metabolism and excretion (expressed as a concentration–time relationship), with the consequent drug response (expressed as the concentration–effect relationship) to describe and predict drug exposure and response	[30, 53–55]
Sheiner’s learn and confirm paradigm	The paradigm of clinical drug development where each phase is designed for distinct purposes, i.e., learning and confirming. Phase I involves learning about general pharmacokinetics and tolerability in healthy patients, whereas phase IIA confirms early efficacy in a limited population. This is followed by a decision node, when positive efficacy can provide evidence to justify accelerating development. Phase IIB then involves learning about variations in PK and PD in target populations while phase III confirms safety and efficacy in a large patient population. This paradigm uses the Bayesian view, wherein prior knowledge from each phase is updated with the availability of new information from subsequent phases of trials using appropriate modeling strategies	[9]
Model-based drug development	The paradigm of drug development utilizing modeling and simulation of drug efficacy and safety, and associated uncertainty in these parameters across preclinical and clinical phases to inform decision making. The key components of model-based drug development include using PK/PD, disease models, meta-analysis of drug and competitor treatment effect sizes, trial execution models describing protocol deviations (e.g., dropout and non-compliance), statistical models describing treatment effect, and decision rules that describe the course of action (terminating or accelerating development) after trial completion	[8, 10]
Clinical trial simulations	Modeling disease progression, clinical pharmacology of drugs, patient covariates, and trial protocol deviations to enable efficient and cost-effective clinical trial design and implementation	[56, 57]
Model-based meta-analysis	Model-based meta-analysis is a quantitative tool that enables comparison of interventions, by aggregating efficacy and safety results from numerous clinical trials while accounting for between-study and between-study-arm variability. This approach utilizes non-linear mixed-effect models and allows characterization of dose–response relationships and the impact of covariates and study and dosing characteristics on patient outcome and efficacy	[58]
Disease progression models	Mathematical representations of the time course of a disease status and progression. These models can be empirical (data-driven descriptions of disease process), semi-mechanistic (data driven, but incorporate some knowledge about [patho]physiological and pharmacological processes), or systems biology (incorporate [patho]physiological and pharmacological processes in molecular detail from integration of in vitro, ex vivo, in vivo, non-clinical, and clinical data)	[59, 60]

*PD* pharmacodynamics, *PK* pharmacokinetics

### Current Regulatory Landscape of Pharmacoeconomics and the Need for Early Economic Evaluations

Single-payer healthcare systems such as the UK, where pharmaceuticals are regulated by the National Institute for Health and Care Excellence, require evidence of cost effectiveness in addition to clinical effectiveness for a pharmaceutical to be approved [12]. However, in the USA, the FDA approves drugs based on safety, efficacy, and clinical effectiveness, and drug price and reimbursement decisions are negotiated between pharmaceutical companies and the payer. While health economic evaluations in the USA have largely been beyond the purview of regulatory control, the FDA recently released its guidance document on how drug and medical device companies can communicate healthcare economic information to payers and formulary committees [13]. The guidance document answers questions about the dissemination of effectiveness, safety, and cost-effectiveness data to payers taking approved drugs such that it is unbiased, factual, accurate, and non-misleading, grounded on reliable scientific evidence. With rising healthcare and pharmaceutical costs and a renewed emphasis on value-based care and pricing in the USA, it has become increasingly important for payers to be convinced of the cost effectiveness of the drugs on their formulary. This has led to regulatory agencies recognizing the value of deliberations between drug manufacturers and payers during the pre-approval phase. The European Medicines Agency has initiated regulatory processes to ensure early communication and parallel scientific advice with the European Network for Health Technology Assessment as a part of its “adaptive pathways” approach. This early collaboration between industry, regulators, health-technology assessment bodies, and payers aims to evaluate the evolving evidence pertaining to drug development and reimbursement in an iterative manner, thus providing decision makers with continuous feedback, rather than a single evaluation [14–16]. A similar “Parallel Review Program” was initiated by the FDA to facilitate communication between medical device manufacturers and public and private payers [17]. This type of regulatory oversight can aid in containing the high research and development costs that now make it imperative for pharmaceutical companies to assess the cost effectiveness of pipeline molecules at earlier stages in the drug development process to minimize the opportunity cost spent on compounds with a low return on investment.

The concept of iterative economic modeling alongside early phases of drug development, which can be considered analogous to MBDD, is not new and was initially proposed in the mid-1990s [18–21]. These early analyses provide insights into the identification of potentially unsuccessful candidates and consequently resource allocation for promising candidates, the design of future non-clinical and clinical studies, and setting pricing strategy and reimbursement policies [22–25]. Recent published studies have used empiric models developed alongside early-phase clinical trials to demonstrate cost ineffectiveness [26, 27] and have generated evidence for not taking development forward to larger phase III trials [28]. The applications and methodology used in early economic evaluations have been reviewed extensively by Hartz and John [29]. In the absence of appropriate clinical trials, safety and efficacy inputs obtained from pharmacometric analyses can be used to supplement economic model-building efforts for decision making in a variety of scenarios.

### Synergies between Pharmacometric and Pharmacoeconomic Methodologies to Accelerate Drug Development

Similar to pharmacoeconomic (PE) models that provide a mathematical framework to describe the costs and consequences of a set of alternative interventions, pharmacometric models are mathematical relationships describing the pharmacokinetic (PK) and pharmacodynamic (PD) characteristics of a drug, and the corresponding effect on disease processes, patient characteristics, and outcomes. Pharmacokinetic models delineate the relationship between drug concentration in the body and time, while PD models include a measure of drug effect [30]. In the absence of individual patient-level data to compare new drugs with standard of care, using summary-level data from the quantitative synthesis of published results of competitive products is also an integral component of MBDD, known as a model-based meta-analysis (MBMA) [31]. The MBMA utilizes pharmacological models such as the exposure–response relationship to characterize the effect of the drug, dose, study design, and patient covariates on the clinical outcome [32]. Most PE evaluations (e.g., Markov models) are empirical, obtaining their probabilities of drug efficacy and safety measures from a variety of sources (e.g., randomized trials, systematic reviews, observational studies, real-world data) and do not consider the mechanistic nature inherent of drug action (i.e., pharmacokinetics, pharmacodynamics, disease progression, and real-world scenarios such as adherence) [33]. Thus, the integration of pharmacometric and PE models provides an opportunity to predict future PE outcomes. More specifically, the outcomes obtained from clinical trial simulations utilizing pharmacometric modeling approaches on efficacy and safety as well as the uncertainty surrounding the model parameters can be used as inputs into PE models allowing the quantification of cost effectiveness.

**Fig. 1:**
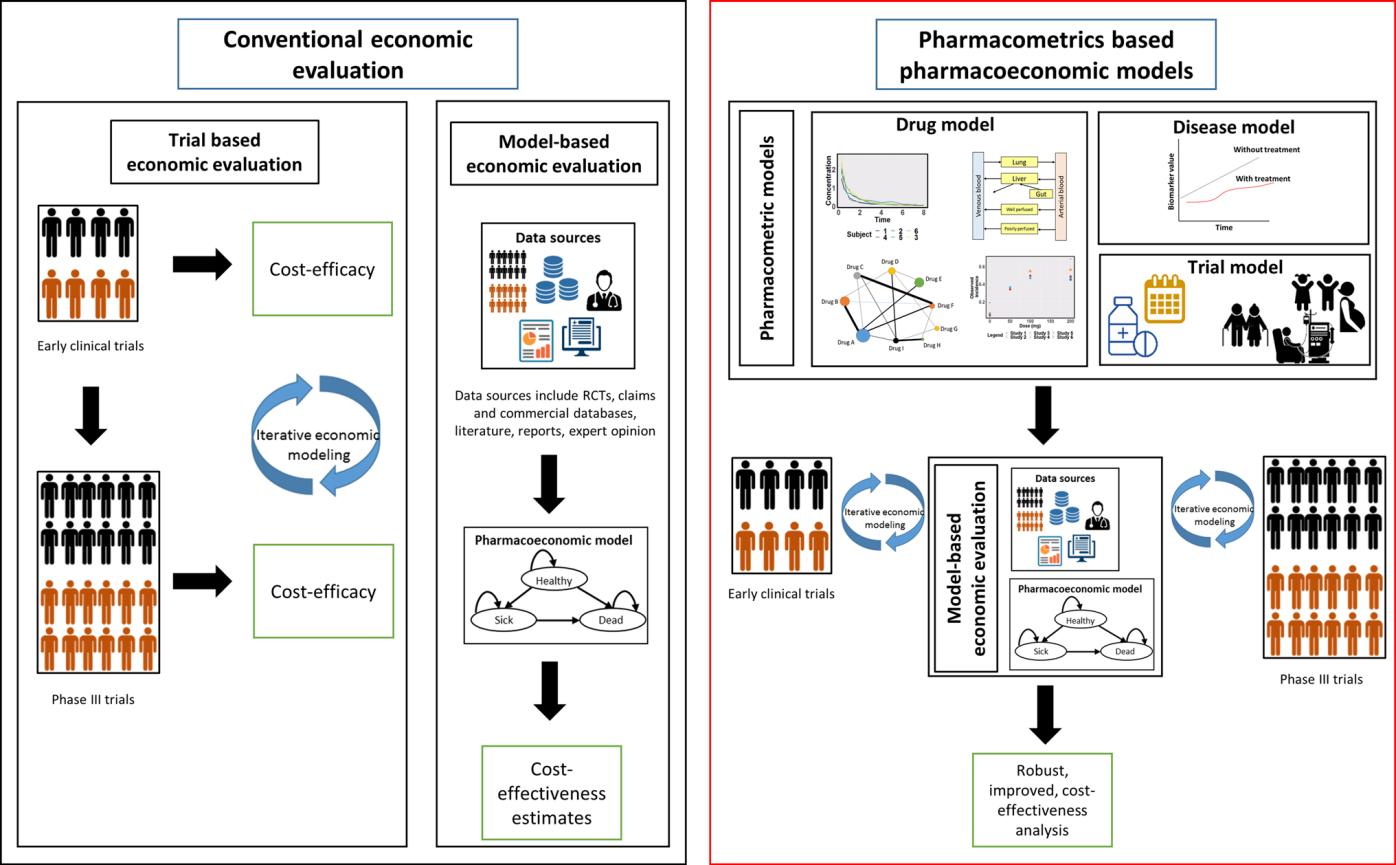
Economic evaluations conducted alongside clinical trials provide estimates of cost-efficacy. Conventional pharmacoeconomic analysis of new drugs are usually performed at the end of phase 3 trials. At this stage, non-establishment of cost-effectiveness might delay the approval and marketing of drugs. Early cost-effectiveness analysis, informed by comparative effectiveness evidence generated from pharmacometric models conducted across the drug development pathway can be informed by the well-established MBDD framework. Drug models include the characterization of concentration-time-effect relationship. Disease progression models describe the relationship between time course of disease and disease-specific biomarkers. Trial model describes protocol-deviations such as medication non-adherence and special populations. These analyses can be used to 1) provide early estimates of cost-effectiveness to support strategic R&D decisions of pipeline drugs (e.g., early termination of uneconomic product, resource utilization) and pricing decisions (e.g., consideration of benefit in value-based pricing). Additionally, they can help model long-term outcomes from surrogate end-points, thus predicting formulations, dosing strategies and patient sub-groups that are likely to show cost-effectiveness, especially in scenarios where conducting clinical trials is not possible. 2) Design efficient and more informative phase 3 trials. 3) Assess the impact of real-world scenarios such as non-adherence, dose adaptation in response to toxicity or public health care utilization patterns and outcomes

Pharmacometrics-derived estimates used as inputs in PE models seek to address the same aims as early-phase economic models, i.e., providing an early estimation of cost effectiveness thus aiding in go/no-go decisions for late-phase development (Fig. 1) [34–44]. As an evaluation of individual patient characteristics that explain PK variability and response is one of the major attributes of pharmacometric models, incorporating these models within PE models can help in the preliminary identification of sub-populations, which are likely to show cost effectiveness with the therapy. Accounting for the exposure–response relationships in pharmacometric models enable the exploration of “what-if” scenarios affecting the cost effectiveness of drugs, e.g., identifying populations that might be susceptible to toxicity or experience therapy failure. The mechanistic nature of pharmacometric models allows for the prediction of outcomes even when clinical data might be sparse, thus facilitating estimation of cost effectiveness for different doses, dosing schedules, and assessing the impact of imperfect adherence to therapy. These methods also aid in the estimation of cost effectiveness in scenarios where conducting clinical trials might be impractical, e.g., comparing therapeutic strategies that might only have a marginal difference in effect, which may necessitate larger sample sizes. Therefore, insights gained from these simulations can provide information for designing efficient clinical trials, thus potentially minimizing late-phase failure.

A sequential approach to generating and synthesizing comparative efficacy data, translating it to real-world effectiveness, and consequently to cost effectiveness can be considered as a natural extension to MBDD [45, 46]. The cross-talk between the fields of pharmacometrics and pharmacoepidemiology to generate, test, and validate a hypothesis iteratively has been found to add value in generating evidence supporting clinical questions [14, 47]. This multi-disciplinary quantitative framework bridging the various subgroups from clinical pharmacology, health economics, outcomes research, regulatory bodies, and payers in drug development has been called the “pharmacology to payer” concept [42]. In this paper, we provide an overview of all published studies that integrate pharmacometrics with pharmacoeconomics and discuss their potential applications in drug development. We also discuss the limitations, challenges, and future opportunities for this inter-disciplinary field of research.

A search of the PubMed database using the keywords “pharmacometric”, “pharmacodynamic”, “pharmacokinetic”, “drug development”, and “clinical trial simulation” along with “economic”, “pharmacoeconomic”, and “cost effectiveness” from 2000 to 2020 resulted in 969 papers. Screening the abstracts for studies that integrated pharmacometrics in PE analyses and hand searching the references of relevant articles yielded 11 publications that fitted the inclusion criteria.

## Pharmacokinetic–Pharmacodynamic–Pharmacoeconomic (PK–PD–PE) Models: History and Current Scenario

A summary of published PK–PD–PE models, along with their applications is shown in Table 2. Most of these studies aimed at demonstrating the proof of concept of the linked PK–PD–PE models and many used hypothetical examples. Therefore, their results should be interpreted in the general context of the feasibility of the approach in various applications of drug development. Of the 11 studies retrieved, nine of these studies were conducted in an academic setting; however, only two were conducted by industry and four had industry collaborators. Therefore, currently, these linked PK–PD–PE models serve as academic exercises demonstrating application of these models on drug development decisions rather than case studies from actual industry projects. The earliest case studies published in 2001 provided descriptions of applications of clinical trial simulations linking PK–PD models with economic models, but did not provide sufficient technical detail. Studies conducted after 2012 expanded on the concept and provided applications with sufficient information to evaluate the implementation and limitations of this approach. Here, we review the case studies categorized by clinical discipline and applications.

**Table 2:** Summary of linked pharmacokinetic-pharmacodynamic-pharmacoeconomic (PK–PD–PE) models published along with their applications

First author (year)	Drug	Pharmacometrics and other models	PE model	General results	Applications
Hughes (2001) [34]	Hypothetical drug for Alzheimer’s disease	Clinical trial (Monte Carlo) simulation using PK–PD models, covariate models, and error models	Decision-analytic model	Estimates of probability of treatment success from clinical trial (Monte Carlo) simulation used as inputs into economic models to obtain distribution of cost-effectiveness estimates	Designing a phase III trial, generating early estimates of cost effectiveness and identifying subgroups in which a drug may provide the most clinical and cost effectiveness
Poland (2001) [35]	Disguised HIV protease inhibitor	Adherence model, PK–PD model	Economic model summarized using net present value metric	Simulations showed sensitivity of formulations to adherence and cost effectiveness of different formulations in various patient sub-groups	Provided data towards development of formulations with suitable PK characteristics for sub-groups of patients
Pink (2012) [36]	Rituximab	Population PK–PD model	Markov model	Simulation- and trial-based estimates showed acceptable concordance for cost effectiveness of rituximab	Informing future value-of-information analysis, pricing decisions, determine impact of protocol deviations (e.g., non-adherence), patient subgroups, dose, and dosing schedules on cost effectiveness
Pink (2014) [38]	Warfarin, rivaroxaban, apixaban, and dabigatran	Population PK–PD model-based clinical trial simulation	Discrete-event simulation model	Apixaban and pharmacogenetics-guided warfarin were cost-effective alternatives to clinically dosed warfarin	Provided cost-effectiveness estimates of various complex dosing algorithms in the absence of comparative clinical trials
van Hasselt (2015) [37]	Eribulin	Disease progression clinical outcome model, PK model, semi-physiological model for neutropenia, ECOG performance score model, covariate model for patient characteristics, dropout model, and dose adaptation model	Cost model, where total cost was a function of absolute cumulative dose received, cost per dose, duration of each adverse event, and cost of adverse event. ICERs calculated for each scenario	Cost effectiveness of various scenarios considering varying dosing regimens, disease progression, patient characteristics, comparators, and adverse event profiles were assessed	Proof-of-concept study provided a framework for integration of disease progression, clinical outcome, toxicities, quality of life, and cost effectiveness to compare various treatment protocols and patient subgroups during early stages of drug development
Slejko (2016) [40]	Hypothetical anti-inflammatory drug for COPD	Model-based meta-analysis	Markov microsimulation model	Impact of hypothetical drug on COPD exacerbations, costs, and QALYs in a patient cohort with varying disease characteristics	Demonstrated the synergistic aspects of MBMA and PE modeling to predict the effect of a drug on patient outcomes and cost effectiveness, which can be used during early-stage PE evaluation of drugs
Kamal (2017) and Wu (2018) [41, 42]	Oseltamivir	Population PK–PD model, susceptible, exposed, infected, and recovered model	Decision tree	Cost effectiveness of oseltamivir in different dosing regimens and transmissibility scenarios	Provides a proof-of-concept using an interdisciplinary framework to estimate the cost utility of an antiviral drug under various pandemic scenarios by linking PK–PD, epidemiological, and economic models during early drug development and allows for conduct of threshold analysis on drug pricing
Hill-McManus (2018) [43]	Lesinurad, allopurinol, and febuxostat	PK–PD model	Markov state-transition model	Impact of non-adherence on cost effectiveness of lesinurad and optimal value-based price was estimated	This model enabled studying how the economic value of new drug treatments depends on pharmacology and adherence to therapy
Hill-McManus (2019) [44]	Febuxostat and hypothetical xanthine oxidase inhibitor analogs	PK–PD model, adherence data using Medication Event Monitoring System	Markov state-transition model	Estimation of impact of increased potency or reduced clearance (drug being more forgiving to missed doses) and its relationship with reimbursement price were quantified	This model provided a structured approach for assessing the value of drugs with desired properties (e.g., more forgiving to missed doses) in early development by providing estimates of pricing options for reimbursement. Allows for justification of their progression through the development process
Wang (2020) [39]	Dabigatran	PK–PD model	Markov microsimulation model	Cost effectiveness of various generics, dabigatran having different systemic exposures, was compared with brand therapy. Generics with high systemic exposure were not cost effective compared with brand references owing to a greater number of bleeding events	The impact of variations in pharmacokinetics-pharmacodynamics into the cost effectiveness of generic drugs with complex pharmacological properties (i.e., with narrow therapeutic indices) was evaluated, which can be informative for regulatory bodies

*COPD* chronic obstructive pulmonary disease, *ECOG *Eastern Cooperative Oncology Group, *HIV* human immunodeficiency virus, *ICERs* incremental cost-effectiveness ratios, *MBMA* model-based meta-analysis*, QALYs* quality-adjusted life-years

### Early Evidence of Potential Applications of Integrated PK–PD–PE Models

The earliest literature proposing a role for early PK–PD data as an input into economic models came from Hughes and Walley [34]. The authors applied the outcomes predicted by a clinical trial simulation utilizing pharmacokinetics-pharmacodynamics to estimate the cost effectiveness of a new drug as compared to the market competitor. This example served to demonstrate how early estimations of cost effectiveness in phase II could help support strategic decisions to inform “go/no-go” decisions for phase II clinical development.

Poland and Wada extended the idea of mechanism-based economic modeling to inform decisions of new formulation development [35]. They illustrated this with the case of whether to pursue a once-a-day regimen for an anti-retroviral drug that was tested in phase II trials as a twice-a-day dosing regimen. The impact of sub-groups of patients responding differently to the drug (experienced patients vs naïve patients) was considered. A Monte Carlo simulation consisting of a drug-disease model combining variability in adherence, pharmacokinetics, and pharmacodynamics was used to simulate a distribution of patient outcomes, assessed by viral load over a varied time period. The economic model used this input of drug efficacy, safety, and usability along with uncertainty parameters (delayed launch date, market share and size, development costs) to ascertain revenue from drug sales into a single value measure, the net present value. The simulations showed that adherence was a key determinant to the therapeutic success of the once-a-day regimen, thus prompting further discovery for a formulation with an extended half-life, minimizing the effect of imperfect adherence. Thus, integration of the components of a drug–disease and economic model could allow for greater insights to be gained for key strategic drug development decisions.

#### Cancer: Semi-Mechanistic and Mechanistic Approaches to Integrate PK–PD–PE Models

After a gap of 11 years in the literature integrating pharmacometric and PE models, Pink and colleagues described a mechanism-based economic modeling approach for rituximab in follicular lymphoma [36]. A published PK and PD model was utilized to simulate progression-free survival data for cohorts of virtual patients receiving rituximab as maintenance and first-line treatment. This simulated progression-free survival served as an input into a cost-effectiveness model of rituximab. The simulated mechanism-based economic analysis was compared to progression-free survival estimates from clinical trial data. There was a reasonable level of concordance between the simulation- and the trial-based estimates, which translated into a consistent impact on reimbursement decisions. This alignment demonstrated the feasibility of this novel approach.

Disease progression and clinical outcome models are disease models that have been used to predict the efficacy of new drugs, based on biomarker response in the early drug development. Using pharmacokinetics, disease progression and clinical outcome, and toxicity models, van Hasselt et al. proposed a proof-of-concept integrated simulation framework for eribulin in the treatment of prostate cancer to predict cost effectiveness [37]. The PK model of eribulin was combined with the disease progression and clinical outcome model describing the dynamics of prostate-specific antigen and overall survival. The main toxicity of eribulin, neutropenia, was modeled using a semi-physiological model describing the time course of neutrophil counts, while Markov transition models were used to describe other adverse events. Real-world situations such as patient dropout and dose changes in response to grade 3 or 4 toxicity were incorporated into the model and simulated for a patient population. Alternative dosing regimens and dose reductions, disease progression criteria, effect of baseline characteristics of patients, safety and efficacy profiles of comparators, and effect of increased toxicity were the various scenarios considered. Costs for treatment and adverse events were assumed and incremental cost-effectiveness ratios were calculated for each scenario. This study provided a comprehensive approach to simulate realistic treatment protocols and relate them to cost effectiveness in early drug development.

#### Anticoagulation: Using PK–PD–PE Models to Evaluate the Cost Effectiveness of Different Treatment Protocols and Generic Drugs

Evaluating the cost effectiveness of novel dosing algorithms is often limited by the large number of potential treatment arms and the small differences in the potential benefits and costs between them, making it difficult to detect differences in outcomes in conventional trials because of sample size constraints. Additionally, most trials are powered to detect differences only in intermediate endpoints and not clinical outcomes. Pink et al. used clinical trial simulations to evaluate the real-world cost effectiveness of clinical and pharmacogenetics-guided warfarin therapy vs newer anticoagulants [38]. A published PK–PD model of warfarin, the output being the daily international normalized ratio values, was used to simulate risk ratios for thromboembolic events using a meta-analysis. A discrete event simulation model was used to simulate clinical events and outcomes. Event rates for the pharmacogenetics-guided cohort obtained from the PK–PD model were compared to the event rates for other interventions obtained from published clinical trials. The authors reported a high probability of pharmacogenetics-guided warfarin and apixaban being cost effective over the traditional approach of international normalized ratio-guided dosing of warfarin. This study provided a framework to use PK–PD models to inform comparative cost effectiveness of different dosing algorithms.

Another novel application of these linked models considers the impact of how variations in pharmacokinetics-pharmacodynamics affect the cost effectiveness of bioequivalent generic vs brand name drugs [39]. Bioequivalence refers to the absence of a significant difference in the rate and extent of absorption of a test and reference product. Two products are considered bioequivalent if the 90% confidence interval of the ratio of the population geometric means of the area under the concentration–time curve and peak plasma concentration fall within 80–125%, i.e., have their average ratios fall within 0.8–1.25 [48]. The authors compare generics of dabigatran with extreme systemic exposure (125% and 80%) and less extreme exposure (112.5% and 90%). The PK–PD model evaluated the difference in exposure and event rates (stroke and bleeding) between generic and brand name dabigatran. These event rates were used as inputs into a Markov model to evaluate the cost effectiveness of the different generic formulations. The study demonstrated that generic formulations while bioequivalent may not be cost effective throughout the admissible range of bioequivalence limits.

#### Respiratory Diseases: Integrating a Model-Based Meta-Analysis and Epidemiology Models in PK–PD–PE Analyses

To facilitate comparisons between interventions and extrapolate relative treatment effects over time when head-to-head trial data are not available, an MBMA and network meta-analysis provide information regarding comparative effectiveness. Slejko et al. used a MBMA model of anti-inflammatory treatments for chronic obstructive pulmonary disease within a Markov microsimulation model to predict exacerbations and compare the cost effectiveness of a hypothetical drug as compared to placebo in the setting of usual therapy in chronic obstructive pulmonary disease [40]. Incorporating MBMA allowed for the prediction of outcome (exacerbations or mortality) between the treatment and placebo groups given individual biomarker forced expiratory volume in 1 s while considering individual-level variables such as exacerbation history, inhaled corticosteroid use, percent of patients washed out of inhaled corticosteroid use, and percent predicted forced expiratory volume in 1 s. This case demonstrated the potential synergy between MBMA and Markov microsimulation to inform a new drug’s value early in the drug development process. Methodological innovations such as a model-based network meta-analysis, which considers the dose–response relationship from multiple agents to predict compound efficacies, have the potential to play to a key role in early drug development and reimbursement decisions [49].

Public health responses to pandemics, depending upon the pathogen transmissibility, have high cost implications from a societal point of view. Models informing pandemic planning have not considered the clinical pharmacology and associated variability of the antiviral drugs on patient outcomes and healthcare resource allocation. Kamal and colleagues proposed a novel proof-of-concept quantitative framework that integrated PK–PD, epidemiological, and economic models to investigate the cost utility of the oral anti-viral agent, oseltamivir in various influenza pandemic scenarios [41, 42]. A published PK–PD relationship between oseltamivir exposure and the time to cessation of viral shedding and alleviation of symptoms was linked to an epidemiological model accounting for the number of susceptible, exposed, infected, and recovered individuals. Simulations of the pandemic for a period of 1 year were performed for scenarios stratified by treatment arm (no treatment, low- and high-dose oseltamivir), percent of population who had an uptake of the drug, and transmissibility. Finally, the infected individuals from each pandemic scenario simulated in the epidemiology model were incorporated into a decision analytic model that was used to conduct a cost-utility analysis. The integrated model was able to simulate the duration of viral shedding, the number of infected individuals, and the cost effectiveness associated with each varying scenario. This study thus proposed an inter-disciplinary framework to assess the economic and epidemiological impact of new drugs using early PK–PD models.

#### Gout: Quantifying Impact of Adherence on Cost Effectiveness

Treatment failure for many diseases results from suboptimal dosing and non-adherence to therapy resulting in, often unnecessary, switching to more expensive second-line agents. Hill-McManus and colleagues aimed to estimate the impact of non-adherence on the clinical and cost effectiveness of the uricosuric agent lesinurad as an add-on treatment in patients non-responsive to allopurinol or febuxostat alone in the treatment of gout, using a PK–PD–PE model [43]. The published PK–PD model was used to model the treatment effect of various scenarios of medication adherence for each of the drug therapy options. A Markov state transition model estimated the lifetime costs and quality-adjusted life-years in a cohort of patients. Apart from predicting the impact of adherence on uric acid target concentration attainment, the model showed how the value-based pricing of lesinurad depended upon adherence. The authors subsequently showed the utility of using PK–PD–PE-linked models along with real-world adherence data (Medication Event Monitoring System) to quantify the maximum reimbursement price of compounds with greater forgiveness to missed doses [44]. This linked model provided a means of studying the implications of favorable properties in drug pharmacology and adherence on the pricing and cost effectiveness of a new drug, a situation of importance while considering the real-world use of a drug.

## Challenges in Implementation of PK–PD–PE Models

The linkage of the disciplines of pharmacometrics and pharmacoeconomics while promising, is still in its infancy, as evidenced by the lack of case studies in the published literature. Although these ideas were first explored in 2001, there remained a considerable gap in the literature until the next publication in 2012. This delay could be reflective of the initial “siloed” nature of these disciplines and the lack of understanding regarding the value of collaboration between these fields; however, as concepts of MBDD are being expanded, methods in which related fields of pharmacoeconomics and pharmacoepidemiology can benefit from inputs from pharmacometrics are being explored [14, 47]. The above reviewed studies had several limitations with regard to the reliability and validity of model assumptions, input parameters, and model integration strategy. Most of the currently published studies only demonstrate the proof of concept of the possible linkage between pharmacometric and PE models and lacked the rigor required for application in decision making for drug development projects. These models relied on published PK–PD models and therefore, model misspecification (model structural uncertainty) remained an important limitation, which restricted extrapolation of the results to situations that were not representative of the population from which the model was developed. Integrating various models may also result in the need to account for multiple parameters, increasing the probability of inaccuracies and bias in the individual parameter estimates (parameter uncertainty). Uncertainty around the parameter estimates owing to high variability and preliminary data from early-phase trials also limit the reliability of the analysis requiring further evidence of the validity of these models. These models involve a linkage of multiple steps, each of which might introduce biases in the final results, for example, extrapolation of short-term endpoints to long-term outcomes [36].

## Future Directions

To address these methodological issues, and ensuring harmonization in terminology, cross-functional teams with relevant collective expertise in clinical pharmacology, pharmacometrics, health economics, statistics, and clinical medicine need to be established [46, 50]. Academic and industry scientists must be encouraged to disseminate applications of PK–PD–PE models in the published literature to foster greater appreciation and acceptance of these methods in the clinical pharmacology and pharmacoeconomics communities. Modelers must develop methodology and qualitative measures to build credibility and assess validity of these models (e.g., sensitivity analysis, bootstrap estimates, and comparing model-derived estimates to actual trial outcomes). To avoid a black-box approach to modeling and to enable the translation of models across disciplines of pharmacoeconomics and pharmacometrics, usage of discipline-agnostic and standard open-source tools (e.g., R) must be encouraged. Greater collaboration between professional membership organizations of both fields will also enable the development of “best practices” towards expanding MBDD to include estimations of cost effectiveness [10]. Collaborative evolution of MBDD will drive greater acceptance from industry, regulatory bodies, and reimbursement agencies to inform pricing and reimbursement decisions.

## Conclusions

Linking pharmacometrics and PE models represents the next frontier for expanding the scope of MBDD [10]. It is therefore imperative that these methods are further explored by researchers in academia and industry to enable greater understanding and acceptance of these methods. Apart from the reductions in cost and improvement in efficiency that may be expected across the drug development continuum, by considering the patient-level variability in the clinical pharmacology of drugs, linked models might also help demonstrate the economic case for precision medicine [51]. Predicting the value of new therapies through merging the fields of pharmacoeconomics and pharmacometrics has the potential to be valuable to the pharmaceutical industry, regulatory bodies, and payers by supporting evidence generation for health technology assessments of new therapies to evaluate comparative safety, efficacy, effectiveness, and cost effectiveness. A multidisciplinary team-based approach through greater collaboration between various stakeholders is required during the research and development process for furthering methodology and applications of these linked models.
